# Influenza A Virus: Host–Virus Relationships

**DOI:** 10.3390/v12080870

**Published:** 2020-08-09

**Authors:** Sunil K. Lal

**Affiliations:** School of Science & Tropical Medicine and Biology Multidisciplinary Platform, Monash University Malaysia, Bandar Sunway 47500, Selangor DE, Malaysia; sunil.lal@monash.edu

We are in the midst of a pandemic where the infective agent has been identified, but how it causes mild disease in some and fatally severe disease in other infected individuals remains a mystery. Similar is the case with most viral diseases, including Influenza. We understand clearly that the severity of disease and the extent of pathogenesis is a direct outcome of the way the infected host reacts to a viral infection. The Influenza A Virus (IAV), just like other viruses, during its course of infection, manipulates and modulates the infected cellular machinery to maintain control and simultaneously convert the cell into a mini factory for viral replication. This becomes a complex host–virus relationship primarily controlled by the viral proteins that are expressed at various time points of the replication cycle, albeit with multifarious changing roles and functions. The host on the other hand, deploys its repertoire of innate immune defenses to suppress and stop the viral infection and replication from progressing. This invisible battle for superiority is what dictates the final outcome of a virus attack on the invaded host cells.

As scientists, many of us try to decipher the molecular mechanisms of disease by understanding these intricate host–viral interactions and their outcomes. From classic single protein–protein interactions to cellular pathways and processes, we try to understand viral strategies that evade host responses. In this Special Issue we have focused on papers pertaining to this subject. 

Besides innate defense mechanisms, the host also exhibits adaptive immunity to restrict viral replication. However, viruses like IAV have multiple strategies to stay cryptic and avoid detection and elimination by such defense mechanisms. Articles by Yun Zhang et al. [1] and Jung and Lee [2] in their reviews have nicely discussed a variety of host proteins that play a pivotal role in triggering antiviral defenses. Later in the review, they have also displayed how IAV has evolved to overcome these host defenses.

Research labs around the globe are devising newer and more robust techniques in order to identify novel ways to perform new antiviral screens. Using live confocal imaging, Anjos Borges et al. [3] have used an innovative Hemagglutinin (HA)-Tetra Cysteine tag reporter IAV in an assay to screen chemical inhibitors of HA translocation. 

Interestingly, our lab [4,5,6,7,8,9,10,11,12,13,14,15] and other labs around the world [16,17,18] have found many target genes that come into the firing line for IAV proteins as vulnerable interaction partners due towhich the host cell becomes vulnerable. Laghlali et al. [19] give a good overview of various cell death pathways and inflammatory pathways that are becoming targets modulation by IAV, leading us to understand new potential antiviral targets in these pathways. Nathan and Lal [20] explain how the 14-3-3 family of proteins play a pivotal role in controlling cellular processes in many DNA and RNA viruses, including IAV. Cheng Fu et al. [21] have neatly discovered the important role of the MDA5 gene in Canine Influenza virus in order to control the NF–kB pathway and hence downregulate cytokine response, and Shipra Sharma et al. [22] have similarly shown elegantly a novel mechanism where IAV stimulates the mTOR pathway and the micro RNA (miR-101) restrains this mTOR expression and viral propagation.

The biggest challenge on our hands, working with IAV and other similar fast-evolving viruses including SARS-CoV2, is that we are dealing with infectious agents that are capable of mutating easily in order to adapt to their new host. This makes combat and control of infection, a never ending and tedious task. Host-adaptive mutations that IAV undergoes make it easily adaptable to a new host, thereby restoring their full activity as described by Chen et al. [23]. This issue is highlighted very nicely by Lutz IV et al. in their review [24], where they have focused on the polymerase gene PA and the ease with which host adaptive mutations occur in it. Interestingly the PA-X protein that is expressed from within the PA gene is believed to have immunomodulatory functions and is quite conserved amongst many IAV strains; but its activity varies between viruses specific for different hosts, suggesting that PA-X may have a role in host adaptation. This review discusses the most recent studies of host adaptive mutations in the PA gene that modulates polymerase activity and PA-X function. It is interesting and noteworthy that although host-adaptive mutations are common in IAV, these changes seldom alter their host proteins interactions, since these interactions mostly are an integral part of the infection and replication cycle of the virus.

With this collection of select publications via a Special Issue on host–viral relationships in IAV, we intend to do our part to contribute to collective information; one more drop in the ocean of information that is already out there on this subject. 
